# Microbiome-derived bacterial lipids regulate gene expression of proinflammatory pathway inhibitors in systemic monocytes

**DOI:** 10.3389/fimmu.2024.1415565

**Published:** 2024-06-26

**Authors:** Saki Mihori, Frank Nichols, Anthony Provatas, Alyssa Matz, Beiyan Zhou, Christopher N. Blesso, Hunter Panier, Lauren Daddi, Yanjiao Zhou, Robert B. Clark

**Affiliations:** ^1^ Department of Immunology, UConn Health, Farmington, CT, United States; ^2^ Department of Periodontology, UConn Health, Farmington, CT, United States; ^3^ Center for Environmental Sciences and Engineering, Institute of the Environment, University of Connecticut, Storrs, CT, United States; ^4^ Department of Nutritional Sciences, University of Connecticut, Storrs, CT, United States; ^5^ Department of Medicine, UConn Health, Farmington, CT, United States

**Keywords:** microbiome, innate immunity, inhibitors, bacterial lipids, TLR, proinflammatory pathways, gene expression

## Abstract

How the microbiome regulates responses of systemic innate immune cells is unclear. In the present study, our purpose was to document a novel mechanism by which the microbiome mediates crosstalk with the systemic innate immune system. We have identified a family of microbiome *Bacteroidota*-derived lipopeptides—the serine-glycine (S/G) lipids, which are TLR2 ligands, access the systemic circulation, and regulate proinflammatory responses of splenic monocytes. To document the role of these lipids in regulating systemic immunity, we used oral gavage with an antibiotic to decrease the production of these lipids and administered exogenously purified lipids to increase the systemic level of these lipids. We found that decreasing systemic S/G lipids by decreasing microbiome *Bacteroidota* significantly enhanced splenic monocyte proinflammatory responses. Replenishing systemic levels of S/G lipids via exogenous administration returned splenic monocyte responses to control levels. Transcriptomic analysis demonstrated that S/G lipids regulate monocyte proinflammatory responses at the level of gene expression of a small set of upstream inhibitors of TLR and NF-κB pathways that include *Trem2* and *Irf4*. Consistent with enhancement in proinflammatory cytokine responses, decreasing S/G lipids lowered gene expression of specific pathway inhibitors. Replenishing S/G lipids normalized gene expression of these inhibitors. In conclusion, our results suggest that microbiome-derived S/G lipids normally establish a level of buffered signaling activation necessary for well-regulated innate immune responses in systemic monocytes. By regulating gene expression of inflammatory pathway inhibitors such as *Trem2*, S/G lipids merit broader investigation into the potential dysfunction of other innate immune cells, such as microglia, in diseases such as Alzheimer’s disease.

## Introduction

The microbiome is believed to play a critical role in a wide range of normal and pathological conditions including normal immune responses, autoimmune diseases, infectious diseases, response to cancer therapy, and pregnancy (1–7). While there have been many studies documenting the association of specific microbiome compositions with diseases and physiologic conditions, the mechanisms underlying these associations remain unclear. One focus of investigation is the identification of potential soluble mediators involved in the crosstalk between the microbiome and systemic niches such as the immune system and the nervous system. Relatively few studies have investigated potential microbiome-derived soluble mediators that crosstalk with the systemic innate immune system compared to those studies investigating effects on the adaptive immune system. Studies investigating microbiome mediators of systemic innate immunity include a documentation of the effects of peptidoglycan on systemic neutrophils (8) and the effects of NOD ligands on the survival and function of systemic monocytes (9). The goal of identifying mechanisms underlying this crosstalk has significant potential for both understanding pathological processes and manipulating the microbiome for therapeutic advantage (10).

Since 2013, our laboratory has studied a family of microbiome bacteria-derived molecules that potentially play a role in mediating the microbiome’s regulation of systemic innate immunity. These molecules are bacterial serine-glycine lipodipeptides (S/G lipids), molecules produced exclusively by bacteria of the phylum *Bacteroidota* (11). Structurally, the dominant species within each S/G lipid class are composed of at least two odd carbon acyl chains bound either to serine and glycine residues or to glycine alone (11). Importantly, the acyl chains contain iso-branched ends, indicating that the S/G lipids are of bacterial origin, and this feature facilitates the identification and quantification of these lipids separately from lipids of mammalian origin using multiple reaction monitoring (MRM) mass spectrometry (11). Currently, there are five known members of this lipid family with Lipid 654 (L654) being the most studied (12). Importantly, S/G lipids, largely as a result of their iso-branched acyl chains, are functional ligands for Toll-like receptor 2 (TLR2) and signal through the TLR2/TLR6 heterodimer (11).

Although L654 is a TLR2 ligand, in a 2013 study of serum L654 in patients with multiple sclerosis (MS), we postulated that L654 was functioning as an immune regulator rather than as an immune stimulator (13). Based on studies of TLR tolerance largely done *in vitro* (14, 15), we further postulated that L654 might normally regulate the level of responsiveness of systemic innate immune responses *in vivo* by gaining access to the systemic circulation and mediating chronic TLR2 signaling that maintains functional levels of feedback signaling inhibitors (16). We subsequently published proof-of-concept studies demonstrating that increasing systemic TLR2 tolerance by administering low-dose exogenous L654 or Pam2CSK_4_ (P2C; a commercially available TLR2 agonist) to mice resulted in the following: (1) significant attenuation of disease severity in a model of MS, experimental autoimmune encephalomyelitis (EAE) (17); and (2) significant enhancement in murine central nervous system remyelination following cuprizone-mediated demyelination (18). Finally, we also documented that a significant percentage of patients with MS, when compared to healthy controls, showed enhanced TLR2-stimulated proinflammatory responses (19). While these studies established the potential for L654 and other S/G lipids to regulate systemic innate immunity, they did not directly address the role of the microbiome in mediating this mode of innate immune regulation. Using a murine model, the goal of our present study was to document the role of the microbiome in this mode of immune regulation and to develop a precision approach for harnessing the microbiome to regulate systemic innate immunity.

## Materials and methods

### Mice

Eight- to 10-week-old female C57BL/6 mice were obtained from The Jackson Laboratory (Bar Harbor, ME). Mice were fed normal rodent chow and were housed in groups of three to five per cage. All mice were maintained under specific pathogen-free conditions in accordance with the guidelines for the Center for Comparative Medicine at the University of Connecticut Health Center. All procedures were performed under Institutional Animal Care and Use Committee-approved protocols.

### Reagents

Vancomycin was purchased from Sigma-Aldrich (St. Louis, MO). Purified Pam2CSK_4_ (Pam2Cys; P2C) was purchased from InvivoGen (San Diego, CA). L654 was derived from *Porphyromonas gingivalis* (American Type Culture Collection 33277 type strain), purified as previously described (11), and generously gifted by Dr. Frank Nichols. EasySep Mouse Monocyte Isolation Kits were from STEMCELL Technologies (Vancouver, BC). The RNeasy Extraction kit was purchased from Qiagen (Venlo, Netherlands).

### Antibiotics

Vancomycin (2 mg total/mouse/day) (Sigma-Aldrich, St. Louis, MO) (or water control) was given as 1 mg via oral gavage twice per day for 6 days. Mice were then “rested” without antibiotics for 6 to 8 days.

### S/G lipid derivation and analysis

#### Fecal S/G lipids

Fecal pellets were obtained from mice after gavage and 6, 8, and 9 days of rest and the fecal pellets were weighed. Using a modification of the Bligh–Dyer technique (20), each fecal pellet was mashed in 6 mL of PBS (Gibco), and five chloroform derivations were run to extract total lipid from the sample. The total S/G lipid family (L342, L430, L567, L654, and L1256) content was identified and quantitated using ultra-performance liquid chromatography coupled with tandem mass spectrometry (UPLC-MS/MS) in MRM mode. Results were standardized to 25 mg of initial pellet weight.

#### Plasma S/G lipids

Blood samples, obtained by cardiac puncture with EDTA-coated syringes, were pooled from five to six mice after gavage and 0, 6, or 8 days of rest. Plasma was derived from these blood samples and frozen. Using a modification of the Bligh–Dyer technique (20), total lipid from approximately 2 mL of plasma was extracted with four chloroform derivations. The total S/G family was identified and quantitated using UPLC-MS/MS in MRM mass mode. The results were standardized to 1 mL of plasma.

### *Ex vivo* stimulation and analysis of cytokine production of splenic monocytes

Splenocytes were derived from mice after vancomycin or water gavage and variable number of rest days. To ensure that sufficient splenic monocytes were derived, prior to monocyte derivation, splenocytes from two spleens were combined from mice of the same cohort. Splenic monocytes (CD11b+/CD3e-/CD45R-/CD117-/Ly-6G-/NK1.1-/Siglec F-/SSC low) were isolated using magnetic bead negative selection (STEMCELL Technologies). A total of 2 × 10^4^ splenic monocytes were cultured in round-bottom 96-well plates in RPMI and 10% FCS and stimulated with either 30 ng/mL, 10 ng/mL, or 1 ng/mL of P2C for 18 h. Two wells were established for each concentration of P2C tested.

Supernatants from individual 96-well cultures of splenic monocytes stimulated with P2C for 18 h were initially frozen and subsequently analyzed for TNFα and IL-6 concentration (pg/mL) using Luminex multiplex assays (MilliporeSigma, Burlington, MA).

### Administering exogenous L654

After a 6-day gavage with water or vancomycin, mice received injections of PBS (vehicle control) or L654 (2.5 or 5 μg/injection) retro-orbitally. These injections were given on rest day 0, 2, and 4 or alternatively throughout the rest period.

### 16S rRNA analysis of microbiome composition

Fecal samples were collected from mice after vancomycin or water gavage and variable number of rest days, and stored at −80°C in DNA/RNA Shield (Zymo Research). The following two methods for 16S rRNA analysis were performed:

1) DNA was isolated from mouse stool samples using the ZymoBIOMICS® DNA Miniprep Kit. Amplification of the V4 region of 16S rRNA gene was performed by PCR using a 515F forward primer for all samples and a unique bar-coded 806R reverse primer for each individual sample, as previously performed (21). Amplified PCR products were sequenced on an Illumina MiSeq platform using the MiSeq Reagent Kit v2 (2 × 250 reads, 500 cycles, Cat #MS-102–2003). Raw sequencing reads were processed through the *DADA2* (v1.22.0) pipeline with default parameters to generate amplicon sequence variants (ASVs). The Silva reference database (version 138.1) was used for taxonomy assignment for each ASV. Raw read counts were converted to relative abundance and analyzed at the phylum level using R (v4.1.0) in the Rstudio interface (v1.4.1106) with the packages *ggplot2* (v3.4.0), *microViz* (v.0.9.0), *magrittr* (v2.0.1), and *phyloseq* (v1.36.0).
2) DNA extraction, PCR amplification, and sequencing of taxonomic marker DNA were performed on fecal samples using the MoBio PowerMag Soil 96-well kit (MoBio Laboratories, Inc) according to the manufacturer’s protocol for the Eppendorf epMotion liquid handling robot. DNA extracts were quantified using the Quant-iT PicoGreen kit (Invitrogen, ThermoFisher Scientific). Partial bacterial 16S rRNA (V4) and fungal ITS2 genes were amplified using 30 ng of extracted DNA as template. The V4 region was amplified using 515F and 806R with Illumina adapters and dual indices [8 base pairs (22)]. The ITS2 region was amplified with ITS3 and ITS4 (23) using the same dual indexing design as the V4. Samples were amplified in triplicate 15-μL reactions using Go-Taq DNA polymerase (Promega) with the addition of 3.3 µg of BSA (New England BioLabs). To overcome inhibition from host DNA, 0.1 pmol primer without the indexes or adapters was added to the mastermix. The PCR reaction was incubated at 95˚C for 3.5 min, with 30 cycles of 30 s at 95.0°C, 30 s at 50.0°C, and 90 s at 72.0°C, followed by final extension at 72.0°C for 10 min. PCR products were pooled for quantification and visualization using the QIAxcel DNA Fast Analysis (Qiagen). PCR products were normalized based on the concentration of DNA from 250 to 400 bp and then pooled using the epMotion 3075 liquid handling robot. The pooled PCR products were cleaned using Omega Bio-Tek Mag-Bind Beads according to the manufacturer’s protocol using 0.8× beads to PCR product. The cleaned pool was sequenced on the MiSeq using the v2 2 × 250 base pair kit (Illumina, Inc). Sequences were processed in Mothur v. 1.36.1 following the MiSeq SOP (22). After demultiplexing and quality checking steps, the sequences were clustered at 97% similarity. Alpha and beta diversity statistics were calculated by subsampling to 10,000 reads per sample. NMS and PERMANOVA were run using the vegan package (24) in R 3.2.0.

### Transcriptomic analysis of splenic monocytes

#### RNA sequencing and data analyses

Following the water, vancomycin, and vancomycin-L654 treatment protocols, splenic monocytes were isolated from mice (*n* = 3 in each group) via the EasySep Mouse Monocyte Isolation Kit (STEMCELL Technologies), yielding 0.2 × 10^6^ cells with a purity of 90%. Total RNA was purified using the miRNeasy Mini Kit (Qiagen, Cat #217084). RNA-Seq libraries were generated using the Illumina TruSeq Stranded mRNA library prep kit by the CGI core and sequenced on Illumina NovaSeq S4 v1.5 for 200 cycles at 10M total paired-end reads of length 100 base pairs. Reads in Fastq files were aligned using HISAT2 v2.2.1 on the GRCm38 index, with SAMtools v1.9 for BAM conversion. A matrix of fragments per kilobase of transcript per million fragments mapped (FPKM) was calculated using Stringtie v2.1.3 with Ballgown on the GRCm38 index.

Data analyses were conducted in R studio v4.2.2. Genes were filtered by a minimum detection frequency of 1%, yielding 24,303 unique genes expressed in at least one sample. For gene expression visualization, the filtered FPKM matrix was log2-transformed with a pseudocount of +1, normalized by quantiles using the NormalizeBetweenArrays function from the *limma* v3.54.2 R package, and expression *z*-score was calculated by mean(gene-expression)/standard deviation (gene-expression).

#### Differential expression

FPKM data were converted to gene count data matrix using the *tximport* v1.26.1 R package. Gene counts were normalized and tested by Wald test using the *DESeq2* v1.38.3 R package. Wald test *p*-value was adjusted using the Benjamini and Hochberg method. Differentially expressed genes (DEGs) were called by an adjusted *p*-value of ≤0.05 (*n* = 962).

#### Gene ontology and pathway analysis

Gene ontology for downregulated and upregulated DEGs was performed using the DAVID Informatics Resource v6.8 (25, 26). For Pathway and Upstream Regulator analysis, the DEGs’ log2 fold change was input to Ingenuity Pathway Analysis [Qiagen (27)]. Pathways and Upstream regulators were filtered by right-tailed Fisher’s Exact Test *p*-value ≤0.05 and an absolute Activation *z*-score ≥1. GO terms query for inhibitors of TLR or NFκB signaling in the MGI Gene Ontology database (http://www.informatics.jax.org/mgihome/GO/project.shtml) returned nine GO terms: GO:0034122, GO:0034136, GO:0032088, GO:0008384, GO:0007249, GO:0007252, GO:1903719, GO:1903720, and GO:1903721. Genes from these terms were combined and examined for expression patterns in our dataset.

Transcription factor (TF) enrichment analyses were performed using ChEA3 (ChIP-X Enrichment Analyses Version 3) tools [*maayanlab.cloud/chea3* (28)]. Output TFs were filtered for interactions mined from the ENCODE project with ChIP-Seq datasets.

### Statistical methods

All data are expressed as mean ± standard error of the mean (SEM). The normality of all datasets was determined by the Shapiro–Wilk normality test. Parametric datasets were analyzed using Student’s *t*-test or two‐way analysis of variance (ANOVA) with *post-hoc* Tukey’s test. Non‐parametric datasets were analyzed using Mann–Whitney *U* test or by Kruskal–Wallis with Dunn’s multiple comparisons test. The statistical analyses were performed using GraphPad Prism version 9 software and *p* ≤ 0.05 was considered statistically significant.

## Results

### Vancomycin oral gavage significantly decreases total fecal and plasma S/G lipids

In preliminary studies, we focused on developing an oral antibiotic protocol that would lower fecal levels of L654. The antibiotics ampicillin, vancomycin, neomycin, and metronidazole were initially tested as a four-antibiotic cocktail. We found that when mice were gavaged for either 6 or 9 days with the four-antibiotic cocktail, the level of S/G lipids in fecal samples were significantly decreased compared to samples from water-gavaged mice. Similarly, when mice were gavaged with either vancomycin or metronidazole alone, the level of S/G lipids in fecal samples was also significantly decreased compared to samples from water-gavaged mice (data not shown). Vancomycin was subsequently chosen as the best candidate based on its ability to lower fecal levels of total S/G lipids and its lack of systemic absorption after oral gavage (29, 30). Although vancomycin is known to primarily target Gram-positive organisms, it has been documented that vancomycin can decrease *Bacteroidota* in the microbiome, potentially through indirect effects (31). In our preliminary studies, we chose to follow levels of total fecal S/G lipids rather than characterizing microbiome composition using 16S rRNA analysis. Microbiome composition was characterized in later aspects of our study. The total fecal S/G lipids analyzed by MRM mass spectrometry were L654, lipid 342 (L342), lipid 430 (L430), lipid 567 (L567), and lipid 1256 (L1256).

In our initial studies, we gavaged female C57BL/6 mice with water (control) or vancomycin twice per day (2 mg of total vancomycin/mouse/day). Mice were gavaged for either 4, 5, 6, or 9 days, and on the day following the last gavage, mice were bled for plasma samples (4-, 6-, and 9-day gavages) and fecal pellets were obtained and frozen prior to MRM mass spectrometric analysis (4-, 5-, and 9-day gavages). Plasma from five to six mice from each cohort (water or vancomycin) and at each time point were pooled to provide sufficient lipids for accurate MRM mass spectrometric analysis.

**Figure 1:**
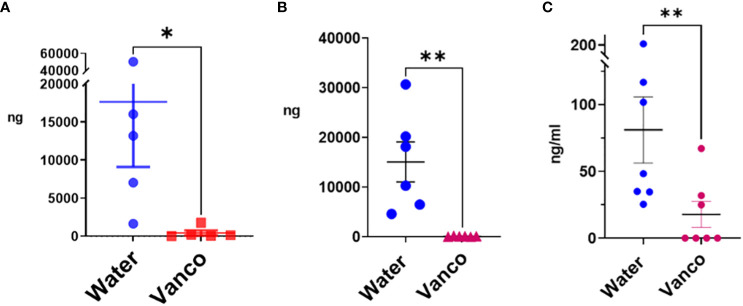
Total S/G lipids in fecal pellets and plasma—without rest period after vancomycin. Total S/G lipids from mice orally gavaged with vancomycin (Vanco) or water (control) for **(A)** fecal S/G lipids; 4- and 5-day gavage; **(B)** fecal S/G lipids; 9-day gavage; **(C)** plasma S/G lipids; 4-, 6-, and 9-day gavage. Each point represents total S/G lipids (ng S/G lipid/25 mg fecal pellet) from one fecal pellet **(A, B)** in which total S/G fecal lipid levels were normalized per 25 mg of fecal pellet, or total S/G lipids/mL of plasma from samples of plasma pooled from five to six mice from the same experiment **(C)**. Data analyzed by Mann–Whitney *U* and expressed as mean ± SEM. *p ≤ 0.05, **p ≤ 0.01.

The 4-, 5-, and 9-day gavage resulted in a significant decrease in total fecal S/G lipids in vancomycin versus water-gavaged mice (**Figures 1A, B**). Similarly, total plasma S/G lipids from 4-, 6-, or 9-day gavaged mice were significantly decreased in the vancomycin versus water-gavaged mice (**Figure 1C**). From these studies, we concluded that oral gavage with vancomycin for as few as 4 days was sufficient to significantly reduce fecal S/G lipids and that this was also associated with a significant reduction of plasma S/G lipids. These early results suggested that a major source of plasma S/G lipids was the GI microbiome.

### Vancomycin oral gavage fails to enhance systemic TLR2 responses

To test whether decreasing microbiome *Bacteroidota* and plasma S/G lipid concentration would result in enhanced systemic innate responses, mice were gavaged with either water or vancomycin for 4 days, and the following day, mice were sacrificed and splenic monocytes were derived. Splenic monocytes were cultured with P2C and culture supernatants were harvested at 18 h and analyzed by Luminex multiplex assay for concentrations of IL-6 and TNFα. Results of these studies demonstrated that despite decreasing the fecal and plasma S/G levels, the 4-day vancomycin gavage protocol used did not enhance, but rather decreased the production of IL-6 and TNFα in response to TLR2 stimulation (data not shown).

Similar to Kolypetri et al. (9), these results suggested that the 4-day gavage with vancomycin resulted in a microbiome unable to support normal numbers and function of systemic monocytes. With the goal of treating the microbiome with antibiotics to decrease levels of *Bacteroidota* (and thus S/G lipids) and yet allowing for a microbiome that was sufficient to support numbers and function of systemic monocytes, we added a rest period after vancomycin gavage. Ubeda et al. reported that while many microbiome phyla, including *Bacteroidota*, were significantly decreased by a 7-day treatment with oral vancomycin, after a 2-week rest period without antibiotics, the microbiome was repopulated by many of the original phyla, but *Bacteroidota* remained significantly decreased (31).

### Fecal S/G lipids

**Figure 2:**
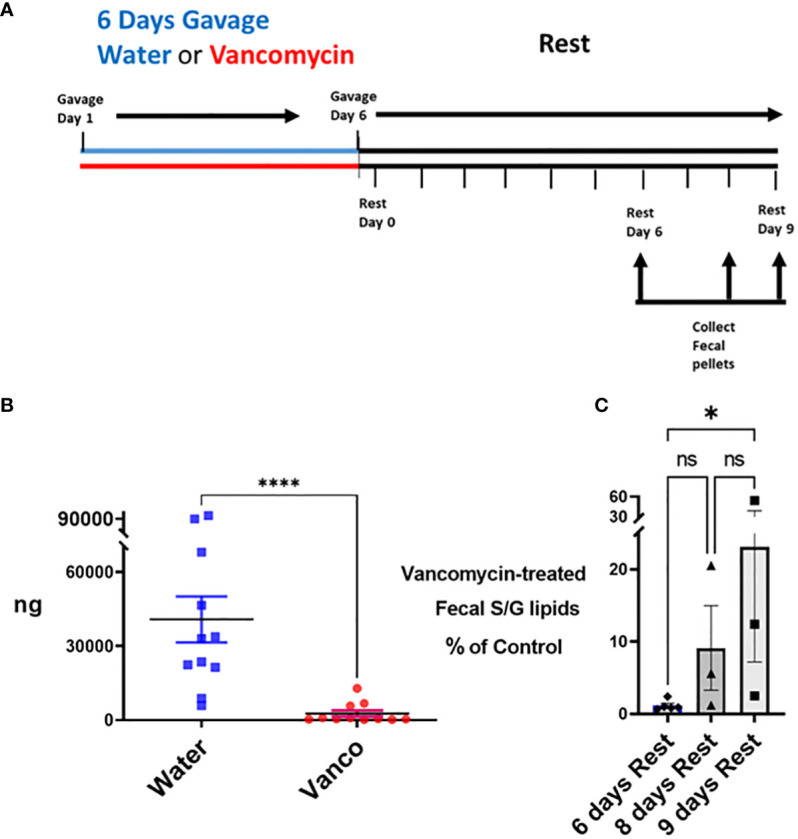
Total S/G lipids in fecal pellets—with rest period after vancomycin. **(A)** Timeline: Mice were gavaged for 6 days with vancomycin (Vanco) or water (control) followed by a no-antibiotic rest period of 6 (*n* = 5 mice), 8 (*n* = 3 mice) or 9 (*n* = 3 mice) days. **(B)** Fecal pellets were collected from these water-gavaged (control) mice and vancomycin-gavaged mice at days 6, 8, and 9 of the rest period. Each point represents total S/G lipids (ng) obtained from one fecal pellet with results normalized per 25 mg/fecal pellet. Data analyzed by Mann–Whitney *U* and expressed as mean ± SEM. **(C)** Total fecal S/G lipids per 25 mg of pellet obtained from vancomycin-treated mice expressed as a percentage of the total fecal S/G of the corresponding water-treated control mice. Data analyzed by Kruskal–Wallis with Dunn’s multiple comparisons test. NS, not significant; *p ≤ 0.05, ****p ≤ 0.0001.

We next tested approaches in which the number of days of gavage and the number of days of rest were varied. We found the most efficient approach to be a 6-day gavage with vancomycin (2 mg total/mouse/day; split between a morning and afternoon gavage), followed by a rest period of 6–9 days in which mice sat undisturbed in their normal housing and no antibiotics were administered (**Figure 2A**). The effect of this approach on fecal S/G lipids was tested first. In mice rested for 6–9 days, the levels of total fecal S/G lipids in vancomycin-gavaged mice were significantly less than those in water-gavaged mice (**Figure 2B**). Furthermore, there appeared to be a trend of less of a decrease in total fecal S/G lipids in vancomycin-treated mice rested for 9 days versus mice rested for 6 days (**Figure 2C**). We interpreted these results to suggest that the proportion of *Bacteroidota* in the microbiome remained low throughout the rest period but evidenced a slow return apparent by day 9 of rest.

### Microbiome composition

**Figure 3:**
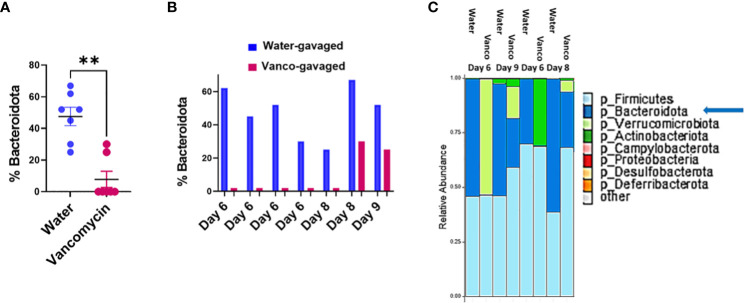
Percent *Bacteroidota* in microbiome. Fecal samples were obtained from mice orally gavaged for 6 days with either vancomycin or water (control), then rested without antibiotics for 6–9 days. DNA was extracted from fecal samples and 16S rRNA was sequenced and processed using two approaches as outlined in Methods. Results are combined from both methodological approaches. **(A)** Percent *Bacteroidota* based on 16S rRNA analysis in fecal samples from mice gavaged with water or vancomycin for 6 days and then rested for 6–9 days; (6-day rest, *n* = 4; 8-day rest, *n* = 2; 9-day rest, *n* = 1). Data analyzed by Mann–Whitney *U* and expressed as mean ± SEM. **(B)** Percent *Bacteroidota* based on 16S rRNA analysis in examples of individual fecal samples rested for 6, 8, or 9 days. **(C)** Relative abundances of major phyla comprising the microbiome in water- and vancomycin-gavaged mice at rest days 6, 8, and 9. **p ≤ 0.01.

To confirm our conclusions based on the fecal S/G lipid studies, we next analyzed microbiome compositions using 16S rRNA analysis of fecal samples obtained from water- and vancomycin-gavaged mice at days 6, 8, and 9 of the rest period. 16S rRNA analysis demonstrated that the proportion of *Bacteroidota* in fecal samples from vancomycin-treated mice were significantly reduced compared to water-gavaged control mice (**Figure 3A**). This decrease in microbiome *Bacteroidota* was true for mice rested for 6, 8, or 9 days (**Figure 3B**). The average proportion of *Bacteroidota* in the 6-, 8-, and 9-day water-gavaged fecal samples was 48% (range, 25%–67%) while the average proportion of *Bacteroidota* in the vancomycin-gavaged samples during the same rest days was 8% (range, 0%–30%). In the vancomycin-gavaged mice, five of seven fecal samples had 0% *Bacteroidota*, with the two higher vancomycin-treated values being seen in the day 8 and day 9 samples (**Figure 3B**). These results are consistent with the pattern of total S/G lipid noted in the fecal samples from the water- and vancomycin-gavaged mice and confirm the relationship between microbiome S/G lipid levels and proportions of *Bacteroidota* in the microbiome. Examples of the major phyla comprising the microbiome in control and vancomycin-gavaged mice at rest days 6, 8, and 9 are depicted in **Figure 3C**.

### Plasma S/G lipids

**Figure 4:**
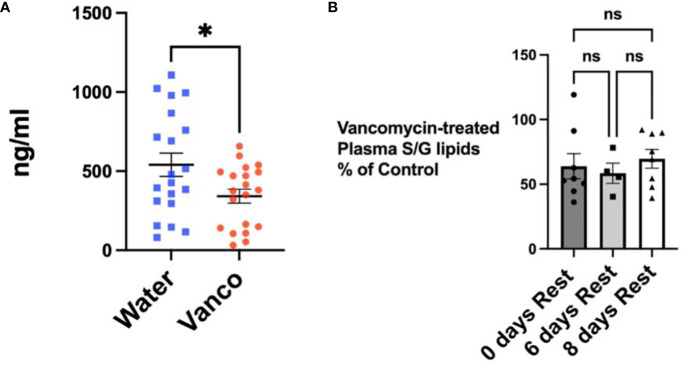
Total S/G lipids in plasma—with rest period after vancomycin. **(A)** Total plasma S/G lipids from mice orally gavaged for 6 days with vancomycin (Vanco) or water (control) with no rest period (*n* = 8) or followed by a no-antibiotic rest period of 6 days (*n* = 4) or 8 days (*n* = 8). Each point represents total S/G lipids/mL of plasma obtained from plasma pooled from five to six mice. Data analyzed by *t*-test and expressed as mean ± SEM. **(B)** Total plasma S/G lipids from mice gavaged with vancomycin followed by no rest days or followed by 6 or 8 days of rest expressed as a percentage of the total plasma S/G lipid of the corresponding water-treated control mice. Data analyzed by one-way ANOVA with *post-hoc* Tukey’s test and expressed as mean ± SEM. NS, not significant; *p ≤ 0.05.

We next wanted to confirm that the decrease in fecal S/G lipid levels in vancomycin/rest-treated mice was also reflected in plasma S/G lipid concentrations. Mice were gavaged for 6 days and then plasma samples were obtained on rest day 0, rest day 6, and rest day 8. When analyzed as one group, the samples derived from all of the mice studied during the 8-day rest period demonstrated a significant reduction in plasma S/G lipids in the vancomycin-treated mice (**Figure 4A**). Furthermore, there was no significant difference in the percentage reduction in plasma S/G lipids in vancomycin-treated versus control mice when comparing the rest day 0, rest day 6, and rest day 8 samples (**Figure 4B**).

It is of note that the percentage decrease in total plasma S/G lipids after 6 days of vancomycin gavage followed by a rest period was not as large as the decrease detected in fecal S/G lipids (**Figure 4** vs. 2). This suggests that factors such as the volume of distribution, the systemic tissue stores of S/G lipids, and the kinetics of metabolism of the S/G lipids differ between the systemic circulation and feces. It is also possible that other microbiomes, such as that of the oral cavity, may contribute to plasma concentrations of S/G lipids. We previously documented that 9 days of vancomycin gavage, rather than the 6-day gavage used in these studies, resulted in almost complete depletion of plasma S/G lipids (data not shown). This supports the concept that the primary source of plasma S/G lipids is most likely the gastrointestinal microbiome and that the concentrations of plasma S/G lipids are more stably maintained by other factors.

### TLR2 responses

Having determined that gavaging with vancomycin for 6 days followed by a 6- to 9-day rest period results in decreased microbiome *Bacteroidota* and decreased fecal and plasma S/G lipids, we next investigated whether this decrease in S/G lipids, in the context of a replenished microbiome, results in enhanced innate immune responses. Splenic monocytes were derived using magnetic bead negative selection, stimulated *ex vivo* with P2C, and 18-h culture supernatants analyzed by Luminex multiplex assay for IL-6 and TNFα concentrations.

**Figure 5:**
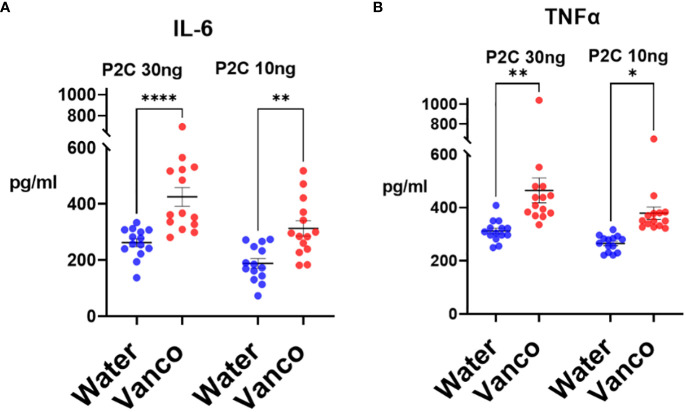
TLR2 responses of splenic monocytes. Mice were orally gavaged for 6 days with either vancomycin (Vanco) or water (control) and then rested without antibiotics for 6–9 days. After the rest days, the mice were sacrificed, and splenic monocytes were derived. Monocytes (2 × 10^4^/well) were stimulated *in vitro* with either 30 ng/mL or 10 ng/mL P2C and culture supernatants were harvested at 18 h and analyzed by Luminex multiplex assays for concentrations of **(A)** IL-6 and **(B)** TNFα. Each point represents the cytokine response of monocytes derived from a combination of two spleens. Data comprise monocytes isolated at rest days 6–9 and analyzed by two-way ANOVA with *post-hoc* Tukey’s test and expressed as mean ± SEM. *p ≤ 0.05, **p ≤ 0.01, ****p ≤ 0.0001.

The decrease in S/G lipids in the context of a replenished microbiome resulted in significantly enhanced P2C-stimulated responses from the splenic monocytes derived from vancomycin-gavaged/rested mice compared to water-gavaged/rested mice (**Figure 5**). This enhanced response was seen for both IL-6 and TNFα and was noted in experiments in which mice were “rested” for 6, 7, 8, or 9 days. These results indicate that in the context of a more normal microbiome, i.e., one regenerated during the rest period, decreased levels of both microbiome *Bacteroidota* and systemic S/G lipids are associated with increased responses of splenic monocytes.

### Administering L654 to antibiotic-treated mice prevents enhanced TLR2 responses

Vancomycin-induced alteration of the microbiome could theoretically result in changes in the availability of numerous potential immune-relevant molecules. We next asked whether administration of exogenous L654 by itself to vancomycin-treated mice could prevent the vancomycin-induced enhanced response and normalize the monocyte TLR2 responses. Initially, we administered either PBS (control) or L654 intravenously (2.5 μg or 5 μg/injection) every day or every other day for the entire 8-day rest period. We initially found that both of these protocols were successful and therefore to simplify the approach, we altered the protocol to administering PBS or L654 (2.5 μg or 5 μg/injection) on rest days 0, 2, and 4 during the 8-day rest period.

**Figure 6:**
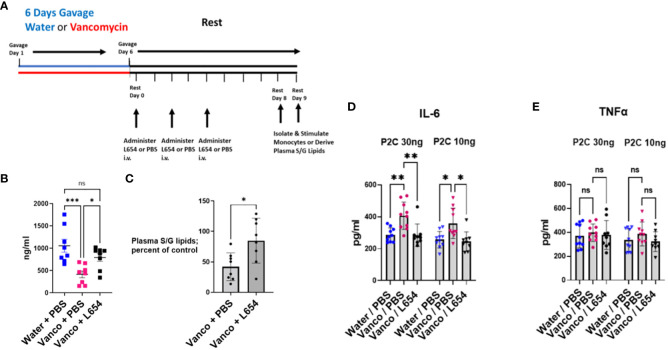
Administration of L654 normalizes TLR2 responses. **(A)** Timeline: Mice were orally gavaged for 6 days with either vancomycin or water (control) and then rested without antibiotics for 8–9 days. PBS or L654 (2.5 μg or 5 μg/injection) was administered intravenously initially every day or every other day for the entire rest period (*n* = 3). Subsequently, the protocol was simplified and PBS or L654 (2.5 μg or 5 μg/injection) was administered on days 0, 2, and 4 during the rest period (*n* = 7). After rest day 8 or 9, splenic monocytes were derived by magnetic negative selection or plasma was obtained by blood pooled from five to six mice. **(B)** To assess changes in plasma S/G lipid levels after administration of L654, we administered L654 intravenously (iv) as described in **(A)** to mice that had been gavaged with vancomycin (Vancomycin + L654). Control cohorts included mice that had been gavaged with vancomycin and injected iv with PBS (Vancomycin + PBS) and mice that had been gavaged with water and injected iv with PBS (Water + PBS). After the 8-day rest period, mice were sacrificed and plasma levels of S/G lipids were assayed. **(C)** Plasma S/G lipid levels of Vancomycin + PBS cohorts and Vancomycin + L654 cohorts expressed as a percentage of control (Water + PBS cohorts) S/G plasma levels. **(D, E)** After the 8- to 9-day rest period, the mice were sacrificed, and splenic monocytes were tested for *ex vivo* response to P2C. Splenic monocytes (2 × 10^4^/well) were stimulated in *ex vivo* culture with either 30 ng/mL or 10 ng/mL P2C for 18 h and the supernatants were analyzed for **(D)** IL-6 or **(E)** TNFα. Data were analyzed by two-way ANOVA with *post-hoc* Tukey’s test and expressed as mean ± SEM. NS, not significant; *p ≤ 0.05, **p ≤ 0.01, ***p≤0.001.

To assess the changes in plasma S/G lipid levels after administration of L654, we administered 5 μg of L654 intravenously on rest days 0, 2, and 4 to mice that had been gavaged with vancomycin (vancomycin/L654 cohort) (**Figure 6A**). Control cohorts included mice that had been gavaged with vancomycin and injected intravenously with PBS (vancomycin/PBS cohort) on rest days 0, 2, and 4 and mice that had been gavaged with water and injected intravenously with PBS (water/PBS cohort) on rest days 0, 2, and 4. After the 8-day rest period, mice were sacrificed and plasma levels of total S/G lipids were assayed. As expected, the levels of plasma S/G lipids in the vancomycin/PBS-injected mice were significantly decreased compared to control levels in the water/PBS cohort (**Figure 6B**). In the vancomycin/L654 cohort, the levels of plasma S/G lipids were significantly elevated compared to those in the vancomycin/PBS cohort. Moreover, the vancomycin/L654 plasma S/G lipid levels were not significantly different from the levels in the water/PBS cohort (**Figure 6B**). When analyzed as a percentage of control plasma levels (i.e., water/PBS cohort), as expected, the S/G lipid levels in the vancomycin/PBS cohort were significantly decreased. The percentages of S/G lipid in the vancomycin/L654 cohort were significantly greater than the percentages in the vancomycin/PBS cohort and not significantly different from those in the control cohort (**Figure 6C**). These results suggest that injecting L654 after vancomycin gavage significantly enhances plasma S/G lipids and returns them to levels approximating those in control mice.

To assess the functional effects of L654 administration, water/PBS, vancomycin/PBS, and vancomycin/L654 cohorts were sacrificed after an 8-day or 9-day rest period and splenic monocytes were tested for *ex vivo* response to P2C. The results (**Figures 6D, E**) represent a combination of studies in which mice were injected using either the every rest day or every other rest day protocol (*n* = 3) or the simplified protocol of injections on rest days 0, 2, and 4 (*n* = 7). We found that administration of exogenous L654 prevented the TLR2-stimulated enhancement in IL-6 production (**Figure 6D**). This indicated that L654 by itself was capable of regulating and normalizing the TLR2 responses. Given the possibility that L654 is not the only microbiome-derived regulatory molecule altered by vancomycin treatment, these results suggest that L654 can function as a “dominant” regulator of these TLR2 responses.

An unusual outcome noted in these L654 administration studies was that although the monocytes from the vancomycin-treated “control” mice gave the expected enhanced IL-6 response (**Figure 6D**), these same monocytes did not show the enhanced TNFα response that was observed in **Figure 5** (**Figure 6E**). As described above, the protocol for these studies involved interrupting the normal period of 8 days of rest. On 3 days within the rest period, the mice were temporarily relocated out of their resting environment to receive an intravenous (retro-orbital) injection of either PBS or L654. Given that this was the only variation from our usual protocol, we believe that the loss of TNFα enhancement together with the persistence of IL-6 enhancement in the same populations of monocytes may be a result of the stress experienced by the mice when their rest period was interrupted three times. Consistent with these findings, TNFα production has previously been reported to be decreased by stress, while IL-6 was not sensitive to the same stress (32). While we cannot be completely certain why the TNFα enhancement was not recapitulated using this protocol, it is clear that the enhanced IL-6 response is strong and normalized by administration of L654, confirming the ability of L654 to regulate TLR2 responses.

### Transcriptomic analysis

To characterize the transcriptomics response to the vancomycin gavage/rest protocol in monocytes, we performed RNA sequencing of primary splenic monocytes isolated from mice in three treatment groups. Three mice from each treatment group were selected after being gavaged with either water or vancomycin for 6 days followed by 8 days of rest (Con and Van groups), whereas the third cohort included mice not only treated with the vancomycin/rest protocol but also injected intravenously with L654 lipid on rest days 0, 2, and 4 (VanLip group). In all three groups, splenic monocytes were collected for RNA after rest day 8 and used for transcriptomic profiling (RNA sequencing) without *ex vivo* stimulation (see **Figure 6A**).

**Figure 7:**
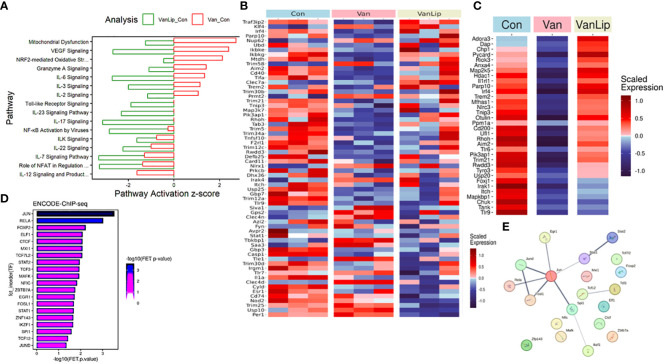
Transcriptomics analyses demonstrate that L654 regulates gene expression of a subset of pathway inhibitors for TLR-NF-κB signaling. **(A)** Pathway analyses of DEGs (DEGs: Van/Con, *n* = 2,822, *p* < 0.05; VanLip/Con, *n* = 3,315, *p* < 0.05) that are sensitive to vancomycin or vancomycin + L654 treatment. **(B)** Regulators of TLR and NF-κB pathways displayed significantly altered expression patterns (*p* < 0.05, *n* = 64) upon Van or VanLip treatment (*n* = 3). **(C)** A set of 34 negative regulators of TLR and NF-κB pathways displayed expression level change sensitive to vancomycin or vancomycin + L654 treatment. The most enriched upstream regulators for the 34 negative regulators of TLR-NF-κB pathways were identified by the ChEA3 program **(D)**, and their connections were displayed using StringDB **(E)**. Expressions of genes in heatmaps were presented with scaled FPKM across all samples.

After the initial data process and normalization (see *Materials and Methods*), we first performed full transcriptomics analyses of all three groups (Con, Van, and VanLip) to detect DEGs (Van/Con, *n* = 2,822, *p* < 0.05; VanLip/Con, *n* = 3,315, *p* < 0.05) followed by gene ontology and pathway analyses using the established database. Interestingly, many well-established pathways contributing to inflammatory responses were activated in the Van group compared to the Con group (Van/Con) but were suppressed after subsequent exposure to L654 (VanLip/Con). These pathways included IL-6, IL-3, NRF2-mediated oxidative stress, and Granzyme A signaling (**Figure 7A**). Another set of pathways were suppressed in both Van and VanLip groups compared to the control group, including ILK, IL-22, IL-7, NFAT, and IL-12. Of note, we also observed that L654 exerted potent suppressive effects on NF-κB and TLR pathways (VanLip/Con), which were relatively unchanged in the Van group (Van/Con) (**Figure 7A**).

Given the critical roles of TLR and NF-κB pathways as direct targets of L654, we examined the dynamic changes of regulators for these pathways using curated terms from databases (KEGG, IPA, and Geneontology.org). We surveyed the pool of genes (*n* = 320) from a total of nine GO terms (see *Materials and Methods*) that included a mix of NF-κB and TLR positive and negative regulators. Interestingly, we detected many genes in this pool that were significantly altered in both the Van or Van/Lip groups (**Figure 7B**). Notably, a set of 34 negative regulators for NF-κB and TLR signaling pathways were suppressed in the Van group but restored in the VanLip group (**Figure 7C**). These L654-sensitive TLR/NF-κB pathway inhibitors included *Trem2, Aim2, Irak1*, and *Irf4* (**Figure 7C**). This suggests that L654 exposure can regulate monocyte responses at the level of gene expression of upstream inhibitors of TLR and NF-κB pathways to establish buffered signaling activation necessary for controlled immune responses in monocytes. This buffering is diminished by vancomycin/rest treatment, resulting in hyper-sensitized cell responses upon activation, and the buffering is restored by administration of L654 (**Figures 5**, **6**).

Next, we performed network upstream regulatory analysis using a TF enrichment tool, ChEA3 (*maayanlab.cloud/chea3*). We focused on the TFs identified by ChEA3 using the ENCODE ChIP-seq datasets, which provide direct molecular interaction detection rather than co-expression analyses. With the input of all 34 negative regulators for NF-κB and TLR signaling that were suppressed in the Van group but restored in the VanLip group, a list of seven TFs received significant enrichment (FET *p*-value < 0.01), including the top two TFs, JUN and RELA (**Figures 7D, E**). These analyses suggest that S/G lipids can directly regulate monocyte sensitivity to stimulation by controlling genes necessary for the negative feedback regulation of inflammatory networks, such as TLR and NF-κB pathways.

Overall, our transcriptomic analysis suggests that the NF-κB and TLR pathways are sensitive to levels of S/G lipids and that this is at least partially mediated through S/G lipid regulation of a subset of NF-κB and TLR pathway inhibitors.

## Discussion

While many studies have documented associations between alterations in microbiome composition and systemic immune-based diseases, little has been documented about mechanisms through which the microbiome regulates the systemic immune system. In this study, we have identified a mechanism through which the microbiome communicates with and regulates the systemic innate immune system. This mechanism involves a specific family of bacteria-derived lipopeptide and lipodipeptide PAMPs (S/G lipids and specifically L654) that access the systemic circulation from the GI microbiome and are postulated to mediate chronic low-level TLR2 stimulation resulting in regulation of innate immune responses. We propose that this regulation is mediated through maintenance of specific levels of signaling feedback inhibitors such as those characterized in TLR tolerance and similar to mechanisms described for “normally educated” immune systems in studies of the hygiene hypothesis (33, 34). Importantly, this mechanism had not previously been well documented for the “internal environment” of the microbiome and we now approached this using well-characterized microbiome-derived molecules and *in vivo* approaches. In the present study, our goal was to document for the first time the direct role of microbiome-derived S/G lipids on regulating systemic innate immune responses.

Given that bacteria of the *Bacteroidota* phylum are the sole producers of L654 and the S/G lipids, we focused on developing an oral antibiotic protocol that significantly decreased microbiome *Bacteroidota*. We initially found that oral gavage of vancomycin, a non-absorbable antibiotic (29, 30), for as few as 4 days successfully decreased fecal and plasma levels of L654, consistent with a decrease in microbiome *Bacteroidota*. However, a 4-day vancomycin treatment was accompanied by a decrease in function of splenic monocytes. Similarly, Kolypetri et al. (9) documented that administering a four-antibiotic mixture to mice disrupted normal microbiome composition and resulted in a decrease in number and function of systemic monocytes. The vancomycin-induced decrease in function of systemic monocytes precluded our ability to detect the innate immune enhancement predicted when we decreased systemic levels of L654. However, Ubeda et al. had previously documented that while 1 week of oral administration of vancomycin resulted in the depletion of many microbiome bacterial populations including *Bacteroidota*, within 2 weeks after stopping vancomycin, a more normal microbiome composition was re-established—except for the persistent depletion of *Bacteroidota* (31).

Based on this published finding, we initiated a protocol consisting of 6 days of oral gavage with vancomycin followed by a rest period without antibiotics for another 6 to 9 days. This protocol proved successful in documenting the significant enhancement in splenic monocyte TLR2-stimulated proinflammatory cytokine production that our hypothesis predicted. Fecal and plasma S/G lipid determination, as well as fecal bacterial 16S rRNA analysis, confirmed that the systemic innate immune response enhancement was associated with a decrease in microbiome *Bacteroidota* and a decrease in fecal and plasma S/G lipid levels. Vancomycin/rest-induced alteration of the microbiome could theoretically result in changes in the availability of numerous as yet unidentified molecules capable of regulating the systemic immune system. We therefore asked whether administering exogenous L654, by itself, could prevent the enhanced innate responses. Indeed, the intravenous administration of L654 to vancomycin-treated mice during the rest period significantly increased the levels of plasma S/G lipids and prevented the enhancement of the TLR2-stimulated responses. This indicated that in the face of numerous potential vancomycin-induced alterations in microbiome-derived immune modulators, L654 acting alone could function as a regulator of microbiome-mediated effects on systemic innate immune responses.

Using transcriptomic analysis, we investigated cellular mechanisms underlying the enhanced splenic monocyte TLR2 responses in vancomycin/rest-treated mice and the reversal of this enhancement after administration of L654. We compared splenic monocytes isolated from three cohorts: control (water-gavaged) mice, vancomycin/rest-treated mice, and vancomycin/rest-treated mice that received L654. Many pathways contributing to inflammatory responses were activated in the vancomycin/rest-treated group but were suppressed after subsequent exposure to L654. Of note, L654 exerted potent suppressive effects on NF-κB and TLR pathways, which were relatively unchanged in the vancomycin/rest-treated mice.

Given the critical roles of TLR and NF-κB pathways as direct targets of L654, we examined the regulators for these pathways. Notably, a set of 34 negative regulators for NF-κB and TLR signaling pathways were suppressed in the vancomycin/rest-treated mice but restored in the vancomycin/rest-treated mice that received L654. This suggests that L654 exposure can regulate monocyte responses at the level of gene expression of upstream inhibitors of TLR and NF-κB pathways to establish buffered signaling activation necessary for controlled immune responses in monocytes. This buffering is diminished by vancomycin/rest treatment, resulting in hyper-sensitized cell responses upon activation, and the buffering is restored by the administration of L654 (**Figures 5**, **6**). Finally, these L654-sensitive TLR/NF-κB pathway inhibitors include those such as *Trem2* and *Irf4* (35–38) that are presently the focus of significant investigation in other cell types and disease-related contexts. This suggests that altering systemic levels of S/G lipids may have translational relevance by altering innate immune inflammatory pathway regulation in contexts such as microglia in CNS disease and T-cell responses in cancer immunotherapy.

In 2013, we found significant decreases in concentrations of blood L654 in patients with MS (13). Many studies of the microbiome in patients with MS have documented a decrease in the percentage of several *Bacteroides* species (39–41). Furthermore, patients with MS with high disease activity have a higher *Firmicutes/Bacteroidota* ratio, increased relative abundance of *Streptococcus*, and decreased *Prevotella* species (*Bacteroidota* phylum) compared to healthy controls and patients with MS with no disease activity (42). In further corroboration, one of the most recent promising findings regarding intestinal dysbiosis in MS is that patients with MS have a lower abundance of *Prevotella* species compared to healthy controls (2, 43) and treatment with disease‐modifying drugs induces an increase in members of *Prevotella* compared with untreated patients (2, 44, 45). The finding of decreased microbiome *Bacteroidota* is consistent with our 2013 study, in which we found significant decreases in concentrations of blood L654 in patients with MS (13).

The microbiome *Akkermansia muciniphila* has been correlated with many systemic effects on host metabolism, favorable outcomes to checkpoint blockade in cancer immunotherapy, and homeostatic immunity (46, 47). In *in vitro* studies, Bae et al. have suggested that a TLR2 agonist—a diacyl phosphatidylethanolamine with two branched chains—recapitulates the immunomodulatory activity of *A. muciniphila* in cell-based assays (47). At low doses *in vitro*, this molecule was shown to have immunoregulatory effects, resetting activation thresholds and responses for immune signaling. The authors suggested that this might provide a mechanism for *A. muciniphila*’s ability to set immunological tone and for its varied roles in health and disease. However, this mechanism has not been further investigated using *in vivo* approaches.

In a recent collaborative study, Millar et al. investigated the relevance of L654 and S/G lipids in an *Ldlr^−/−^* murine model of high-fat diet (HFD)-induced atherosclerosis (48). These investigators documented that feeding these mice with an HFD resulted in decreased microbiome *Bacteroidota* and decreased fecal and serum L654. In addition, they found that intraperitoneal administration of L654 over 7 weeks resulted in hypocholesterolemia, significantly attenuated the progression of atherosclerosis, and reduced liver inflammatory gene expression. Investigating a chow-fed *Apoe^−/−^* murine model of atherosclerosis where “normal” fecal S/G lipid exposure is maintained, 7-week administration of L654 was still found to reduce markers of liver injury but had a weaker effect on atherosclerosis endpoints compared to the HFD-fed *Ldlr^−/−^* model. Overall, this study documented that conditions in which gut microbiome-derived S/G lipids are depleted can exacerbate the development of atherosclerosis and liver injury, whereas correction of such depletion protects from these disorders (48).

The major findings of the present study are as follows: (1) microbiome *Bacteroidota*-derived L654 and the family of S/G lipids act as mediators in the crosstalk between the microbiome and the systemic innate immune responses of splenic monocytes; (2) a specific protocol of oral vancomycin followed by a short rest period results in a microbiome that supports normal innate immune responses but remains depleted in *Bacteroidota*; (3) the vancomycin-induced depletion of microbiome *Bacteroidota* is associated with decreases in fecal and plasma levels of S/G lipids; (4) the decreased plasma levels of S/G lipids is associated with enhanced *ex vivo* splenic monocyte, TLR2-stimulated, proinflammatory cytokine production; (5) despite the potential for vancomycin to induce alterations in an unlimited number of as yet unidentified microbiome-derived immune regulatory molecules, exogenous administration of L654 by itself is sufficient to regulate and inhibit the enhanced innate responses; (6) in contrast to our previous studies in which increasing S/G lipid levels through administration of exogenous L654 resulted in diminished systemic innate immune responses (17, 18), in the present study, we directly confirmed the role of the microbiome in this regulatory paradigm and demonstrated that decreasing systemic S/G lipid levels results in enhanced innate immune responses; and (7) systemic S/G lipids regulate splenic monocyte responses at the level of gene expression of upstream inhibitors of TLR and NF-κB pathways to establish buffered signaling activation necessary for controlled immune responses in monocytes.

Overall, our results suggest that S/G lipids mediate crosstalk between the microbiome and splenic monocytes. By regulating gene expression of inflammatory pathway inhibitors such as *Trem2*, S/G lipids merit broader investigation into the potential dysfunction of other innate immune cells, such as microglia, in diseases such as Alzheimer’s disease (37, 38).

## Data availability statement

Data are available in the manuscript and there are no restrictions on data availability. The RNAseq data were deposited to NCBI GEO. The accession number is GSE269771.

## Ethics statement

The animal study was approved by Institutional Animal Care and Use Committee Office of the Vice President for Research, UConn Health. The study was conducted in accordance with the local legislation and institutional requirements.

## Author contributions

SM: Conceptualization, Data curation, Formal analysis, Validation, Visualization, Writing – original draft, Writing – review & editing, Investigation, Methodology. FN: Conceptualization, Data curation, Formal analysis, Investigation, Methodology, Writing – review & editing. AP: Conceptualization, Data curation, Formal analysis, Investigation, Methodology, Writing – review & editing, Resources, Software. AM: Conceptualization, Data curation, Formal analysis, Investigation, Methodology, Writing – review & editing. BZ: Conceptualization, Formal analysis, Investigation, Methodology, Writing – review & editing, Data curation, Supervision. CB: Conceptualization, Formal analysis, Investigation, Methodology, Writing – review & editing, Visualization. HP: Data curation, Formal analysis, Investigation, Methodology, Writing – review & editing. LD: Conceptualization, Data curation, Formal analysis, Methodology, Writing – review & editing, Validation. YZ: Data curation, Formal analysis, Methodology, Writing – review & editing, Conceptualization, Supervision. RC: Conceptualization, Data curation, Formal analysis, Validation, Writing – review & editing, Funding acquisition, Project administration, Supervision, Visualization, Writing – original draft.
